# In Vivo and In Vitro Analyses of Titanium-Hydroxyapatite Functionally Graded Material for Dental Implants

**DOI:** 10.1155/2021/8859945

**Published:** 2021-04-30

**Authors:** Xinhua Wang, Chengpeng Wan, Xiaoxia Feng, Fuyan Zhao, Huiming Wang

**Affiliations:** ^1^The Affiliated Stomatology Hospital, Zhejiang University School of Medicine, China; ^2^Key Laboratory of Oral Biomedical Research of Zhejiang Province, China

## Abstract

**Purpose:**

The stress shielding effect caused due to the mechanical mismatch between the solid titanium and the surrounding bone tissue warrants the utilization of a mechanically and biologically compatible material such as the titanium-hydroxyapatite (Ti-HA) functionally graded material (FGM) for dental implants. This study is aimed at fabricating a Ti-HA FGM with superior mechanical and biological properties for dental implantation.

**Materials and Methods:**

We fabricated a Ti-HA FGM with different Ti volume fractions (VFs) using HA and Ti powders. Ti-HA was characterized by studying its mechanical properties. Cytotoxicity was examined using a Cell Counting Kit-8 assay and an LDH cell cytotoxicity assay. Scanning electron microscopy was performed on an XL30 environmental scanning electron microscope (ESEM). Alkaline phosphatase (ALP) and transforming growth factor (TGF-*β*1) expressions were quantitatively monitored using enzyme-linked immunosorbent assay (ELISA) kits. The expressions of *TGF*-*β* receptors and ALP genes were measured using real-time polymerase chain reaction. The Ti-HA FGM dental implants were placed in beagle dogs. Microcomputed tomography (CT) and hard tissue slices were performed to evaluate the bone-implant contact (BIC) and bone volume over total volume (BV/TV).

**Results:**

The density and mechanical properties of the Ti-HA exhibited various graded distributions corresponding to VF. Based on the results of the Cell Counting Kit-8 (CCK-8) and lactate dehydrogenase (LDH) assays, the difference in cytotoxicity between the two groups was statistically nonsignificant (*P* = 0.11). The ALP and TGF-*β*1 levels were slightly upregulated. The transcript levels of ALP and TGF-*β*RI were higher in the Ti-HA groups than in the Ti group at 7 days, whereas the transcript levels of TGF-*β*RII exhibited no obvious increase. The BIC did not exhibit significant differences between the Ti and Ti-HA FGM groups (*P* = 0.0504). BV/TV showed the Ti-HA FGM group had better osteogenesis (*P* = 0.04).

**Conclusion:**

Ti-HA FGM contributes to the osteogenesis of dental implants in vivo and in vitro.

## 1. Introduction

Anterior tooth loss affects aesthetics, whereas posterior tooth loss affects mastication. Long-term neglect of this problem may subsequently result in complications such as migration of the adjacent tooth, extrusion of the antagonist, alveolar bone atrophy, and dysfunction of the temporomandibular joint [1, 2]. Dental implants are usually considered the optimum treatment option for tooth loss. Dental implants made of titanium and titanium alloys are considered the gold standard and have been successfully used for a variety of indications such as abutment for removable prostheses, fixed single-tooth reconstructions, and fixed dental prostheses [3].

However, the mechanical mismatch between the solid titanium and the surrounding bone tissue creates a stress shielding effect, complicating the process of osseointegration and bone remodeling. Although their short-term results were satisfactory with a 5-year success rate as high as 90%, the long-term results were still not satisfactory [4]. Thus, a new implant design was deemed necessary.

Literature based on finite element analysis indicated that the geometry and implant material play a major role in reducing the maximum stress and stress shielding effect around a dental implant [2, 5–8]. Hydroxyapatite was coated on a dental implant using a biocompatible and bioactive ceramic [9]. However, the high risk of crack formation at the coating layer and dental implant interface introduces undesirable deficiencies during the manufacturing process. These deficiencies may be improved using functionally graded material (FGM) implants; however, it is challenging to develop biomaterials that are both biologically and mechanically compatible [10].

Titanium (Ti) has exhibited low density, high mechanical strength, and good biocompatibility. However, the elastic modulus values of human bones (cortical bones, 10–20 GPa; cancellous bones, 1.5–2.5 GPa) are considerably lower than those of commercial titanium implants (approximately 110 GPa), which is over ten times that of bone [4]. According to Wolff's law, commercial titanium implants will result in bone resorption and atrophy around the implant, ultimately leading to implant mobility and failure [11].

Hydroxyapatite (HA) is the most extensively studied and widely used developing bioceramic in dentistry due to its biological and crystallographic similarities with hard tissue. Furthermore, it stimulates natural bone formation around the dental implant to promote successful osseointegration [12]. The main use of HA in implantology is as a coating material for titanium implants, and its effect on osteointegration, inflammatory response, and antibacterial activity was assessed [13].

The Ti-HA FGM was successfully developed by using HA in the implant body [14]. It effectively utilized HA and Ti to fabricate a FGM compositional distribution profile. Ti-HA FGM exhibited better biocompatibility and osseointegration than Ti. Although functionally graded titanium scaffolds were fabricated, no biological properties of this material were tested, precluding its use in dental implants due to the fear of adverse effects due to mechanical or biological properties.

The Ti-HA FGM signifies a new class of dental composites. This study is aimed at fabricating a Ti-HA FGM with good biological characterization for dental implantation.

## 2. Materials and Methods

### 2.1. Fabrication of the Ti-HA FGM Dental Implant

**Figure 1:**
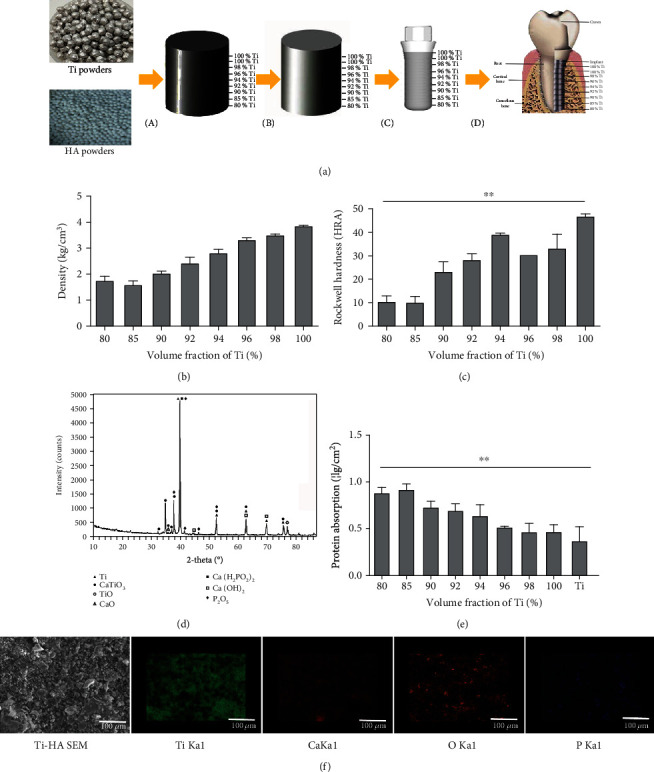
Mechanical characterization of the Ti-HA FGM. (a) (A) HA powders and Ti powders were mixed from top to bottom according to the volume fraction (VF) of Ti, namely, 80%, 85%, 90%, 92%, 94%, 96%, and 98%. (B) Green compacts with graded Ti VF were sintered at 1300°C in an argon atmosphere heated at 10°C/min and cooled at 6°C/min. (C) Cylindrical implants (diameter 4.1 mm, length 10 mm) with graded Ti VFs were fabricated as Ti-HA FGM dental implants through subtractive manufacturing. (D) The Ti-HA FGM dental implant was placed into the mandibular bone. (b) Density of the sintered samples increased with the increase of Ti VF. (c) The HRA value gradually increased from 10 to 47 with an increase in slag loading (*n* = 3, ^∗∗^*P* = 0.0005). (d) XRD pattern of 80% Ti-HA alloy. No additional new phases were observed, indicating that no new phases were formed in the ball milling process. (e) Concentration of adsorbed protein on the material surfaces was measured (*n* = 3, ^∗∗^*P* < 0.0001). (f) Scanning electronic microscopy with energy-dispersive spectroscopy (SEM-EDS): (A) electron micrographs, (B) Ti, (C) Ca, (D) O, and (E) P. The elements were evenly distributed and did not exhibit any specific features.

The materials were fabricated according to a method reported by Chenglin and Zhongda [15]. HA powder (Sigma-Aldrich, St. Louis, MO, USA) and Ti powder (Gamma Technology Development Co., Ltd., Shenzhen, China) were used to fabricate the alloy. Both powders were mixed together according to different volume fractions (VFs) of Ti (80%, 85%, 90%, 92%, 94%, 96%, 98%, and 100%) (Figure 1(a)). Then, we measured the density and Rockwell hardness (HRA) of each material.

Commercially available pure Ti was bought from Baoji Shenghui Titanium Co., Ltd. (Baoji, SX, China). Initially, different Ti-HA mixing ratios were used and the powders were blended by ball milling for 12 h (HORIBA, Kyoto, Japan). Then, the Ti-HA mixture was stacked in a steel die compacting at 20–30 MPa. Finally, the green compacts were sintered at 1300°C in an argon atmosphere at 10°C/min and cooled at 6°C/min in a high-temperature calciner (Xiyuzhuogong, Henan, China).

Figure 1(a) illustrates the step-by-step implant design process. Implants with a 4.0 mm diameter and 10.0 mm length were fabricated in an implant manufacturing company (ZDI, Cixi, Zhejiang, China).

### 2.2. Study of Mechanical Properties

The density of the samples was measured by Archimedes' method. The Rockwell 15T hardness (HRA) of specimens was measured with a 15 kg force. The phase constitution was analyzed by X-ray diffraction (XRD, X-Pert, PRO-MPD, PANalytical B.V.), whereas the chemical composition of the surfaces was analyzed using an XL30 environmental scanning electron microscope (ESEM; FEI Company, OR, USA) and energy-dispersive spectrometry (EDS; OxfordAZtech x-max 80, Oxford Instruments, Oxfordshire, UK). A protein adsorption assay was performed to determine the total area of the material surfaces. After attaining equilibrium in phosphate-buffered saline (PBS; 0.01 M, pH 7.4) for 1 h, the specimens were incubated in 0.1 mg/mL bovine serum albumin (BSA) solution (Sangon Biotech, Shanghai, China) at 37°C for 1 h. Then, the specimens were treated with 0.1 wt% sodium dodecyl sulfate (SDS; Sangon Biotech, Shanghai, China). The concentration of absorbed BSA was measured at 562 nm using the Micro BCA™ protein assay kit (Pierce, IL, USA) in a microplate reader (Thermo Fisher Scientific Inc., MA, USA).

### 2.3. Mechanical Characterization of Ti-HA FGM

The density of the Ti-HA FGM increased with the increase in the Ti VF (Figure 1(b)). The least density was seen when the Ti VF was 80%, and the maximum density was observed when the Ti VF was 100%. The HRA increased from 10 to 47.0 with an increase in the VF of Ti from 80 to 100% (Figure 1(c)). XRD analysis revealed that HA and Ti mainly existed as simple substances, and no reactions between HA and Ti could be detected (Figure 1(d)). The Ti-HA FGM groups showed a higher absorption of BSA compared with the Ti group, implying that the Ti-HA FGM could absorb protein well (Figure 1(e)). EDS analysis of the alloy (Figure 1(f)) demonstrated the presence of Ti, calcium, phosphorous, and oxygen. Thus, the Ti-HA FGM showed increasing density and hardness with increasing Ti VF. The two components existed without reacting or forming a complex, and the composite showed better absorption of BSA than Ti alone.

### 2.4. Cell Growth and Differentiation and Cytotoxicity

Commercially available preosteoblast MC3T3-E1 cells (donated by Dr. Shi Jue, Zhejiang University) were used for the study. Briefly, MC3T3-E1 cells were cultured in modified Eagle's minimum essential medium (Gibco, Invitrogen, USA) supplemented with 10% fetal bovine serum (Gibco, Invitrogen, USA). They were then washed in a 37°C water bath for 2 min and transferred to a 25 cm^3^ culture flask for culture at 37°C in an atmosphere of 5% CO_2_.

Cells were seeded on Ti surfaces at a density of 1 × 10^4^ cells/cm^2^ in triplicate. The cells were studied after 48 h of culture, fixed with 2.5% glutaraldehyde overnight at 4°C, dehydrated through a serial alcohol gradient, and then critical point-dried with liquid CO_2_ (Hitachi, Tokyo, Japan). The samples were observed under an XL30 ESEM (FEI Company, OR, USA).

MC3T3-E1 cells were seeded in 12-well plates containing Ti and Ti-HA disks at a density of 1.0 × 10^4^ cells/well and incubated for 1, 4, 7, and 14 days. The culture medium and unattached cells were removed, and the medium was replaced with a fresh medium every 24 h. The total cell count was obtained by direct cell counting with a Vi-CELL™ XR cell viability analyzer (Beckman Coulter, Inc., USA).

Ti cytotoxicity was assayed by seeding the cells in a Cell Counting Kit-8 (CCK-8) assay containing Ti disks (Longteng Bio-Science, Shanghai, China). Absorbance was read at 450 nm by a microplate reader (BioTek Instruments, Inc., Vermont, USA).

Cells were plated at a density of 9 × 10^2^ viable cells per well in 96-well culture plates and cultured for 24 h in Eagle's minimum essential medium- (EMEM-) based complete medium to allow cell attachment. To evaluate the cytotoxicity of FGM in osteoblasts, MC3T3-E1 cells were exposed to different FGMs and incubated for 48 and 120 h. Then, lactate dehydrogenase (LDH) assay was performed using LDH Cytotoxicity Kit II (Wuhan Amyjet Scientific Inc., China) in accordance with the manufacturer's instructions. All tests were performed in triplicate. The percentage of cytotoxicity was calculated using the following equation: (test sample–low control)/(high control–low control) × 100, where the cells cultured in EMEM plus cell lysis solution represented the high control while those cultured in EMEM alone represented the low control.

### 2.5. Real-Time Quantitative Polymerase Chain Reaction (RT-qPCR)

Protein expression in the medium was detected using enzyme-linked immunosorbent assay (ELISA) kits (Shanghai Lengton Biotech, China). Transforming growth factor (TGF-*β*1) and alkaline phosphatase (ALP) were measured according to the manufacturer's instructions. Changes in the ALP, TGF-*β*RI, and TGF-*β*RII transcriptional levels on different materials were examined with RT-qPCR.  Table 1 lists the primers used for *ALP*, *TGF*-*βRI*, and *TGF*-*βRII*. The cDNA species were synthesized using Promega M-MLV (Promega, WI, USA) in accordance with the manufacturer's instructions. Real-time PCR was performed using SYBR® Premix Ex Taq™ (Takara Biotechnology, Shanghai, China) in accordance with the manufacturer's instructions. Upregulated genes were confirmed using a StepOnePlus Real-Time PCR System (Applied Biosystems, USA). Gene expression levels were normalized to *GAPDH* expression. The relative expression level was calculated by *ΔΔ*CT of the 2^–*ΔΔ*CT^ method [16].

### 2.6. In Vivo Animal Studies

The animal study was approved by the Animal Experimentation Ethics Committee of Zhejiang University. The number of animals was reduced to a minimum according to the “3Rs” (refinement, reduction, and replacement) [17]. All experiments were performed in accordance with the Guidelines to the Care and Use of Experimental Animals. Six 12-month-old beagle dogs (average weight, 11.3 kg) were selected from the Animal Experimentation Center of Zhejiang University.

**Figure 2:**
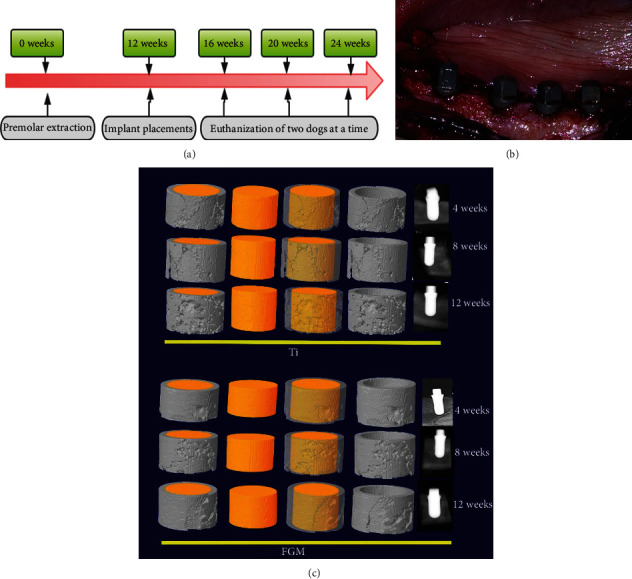
Animal experiment design and micro-CT scan of Ti-HA FGM dental implants. (a) Design of the animal experiment. Implants were placed in the mandibles of beagle dogs 12 weeks after the premolars were extracted. After another 12 weeks of healing, the dogs were sacrificed. (b) Sample perioperative photograph. The dental implants were placed in the healing mandible of the dog. (c) Micro-CT and X-ray analyses were used to evaluate the changes in bone quality on the mesial and distal side of the implant and the peri-implant new bone formation at 4, 8, and 12 weeks after implant placement. At 4 weeks, the Ti-HA FGM dental implants had better bone quality around the implant compared with the Ti group. Up to 12 weeks, more peri-implant new bone formation was observed around the Ti-HA FGM dental implants than around the Ti implants.

All mandibular premolars and first molars were extracted bilaterally during the first surgical session. After 3 months of healing, full-thickness flaps were elevated, and two implants (Ti-HA FGM and Ti implants; 10 mm length, 4.0 mm diameter) were installed on each side of the mandible in the premolar region (Figure 2(b)). All implants were placed slightly subcrestal both buccally and lingually to obtain primary stability as performed in another study [18]. The flaps were sutured to ensure watertight healing. After surgery, each animal received amoxicillin (250 mg/day; Hainan Sanye Pharmaceutical Group Co., Ltd.) and metronidazole tablets (250 mg/day; Xi'an Fenghua Pharmaceutical Co., Ltd.). Finally, the dogs were sacrificed at 4, 8, and 12 weeks. The implant site was removed using a diamond saw.

The biopsies were processed for ground sectioning [19]. Bone regeneration was measured on microcomputed tomography (CT) as the bone volume over total volume (BV/TV; Skyscan 1176, Bruker Micro-CT N.V., Belgium). The bone-implant contact (BIC) ratio was determined at a three-dimensional (3D) level and recorded with Evaluation v6.5-3 software (Scanco Medical, Switzerland). A high-speed precision microtome (Leica 2500E, Germany) was used to obtain serial sections (150 mm) along the axis of the implants. Sections were stained with methylene blue/acid fuchsin, and osteogenesis and bone maturity were assessed under a light microscope (Leica, Germany). Bone mass was determined using the image analysis software (Image-Pro Plus 6.0, Media Cybernetics, Rockville, MD, USA). The parameters (brightness, contrast ratio, and white balance) were constant during the whole histological examination.

### 2.7. Statistical Analyses

Statistical analysis was performed using SPSS for Windows 10 software (IBM SPSS Statistics for Windows, Armonk, NY, USA). The quantitative variables for each sample and implant were expressed as mean ± standard deviation. Qualitative variables were statistically analyzed using one-way analysis of variance (ANOVA), two-way ANOVA, Tukey's test for multiple comparison tests, and Student's *t*-test. *P* < 0.05 was considered statistically significant.

## 3. Results

### 3.1. Cell Growth on Ti-HA FGM in Standard Culture Conditions

**Figure 3:**
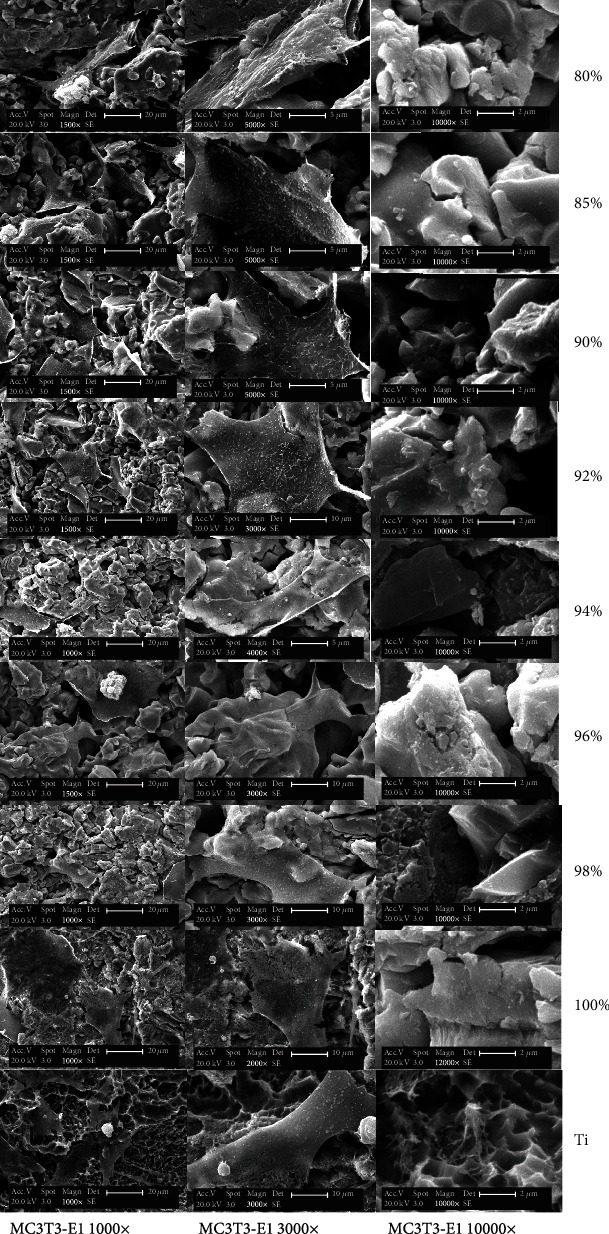
Cell growth in standard culture conditions. SEM micrographs demonstrate the surface topography of the surfaces of the Ti-HA and Ti materials used. MC3T3-E1 cells migrated into the grooves, and many cellular pseudopodia attached to the walls of the grooves in the Ti-HA composite, whereas in the Ti group, cells adhered only to the surface of the material.

SEM pictures showed that the Ti-HA FGM with different Ti VFs had different surfaces compared with Ti (Figure 3). The Ti-HA FGMs composed of Ti with lower VFs had relatively more grooves than those composed of Ti with higher VFs. MC3T3-E1 cells migrated into the grooves and attached to the walls of the grooves with pseudopodia, whereas cells in the Ti group grew only on the surfaces of the material.

### 3.2. Cell Viability on Ti-HA FGM in Standard Culture Conditions

**Figure 4:**
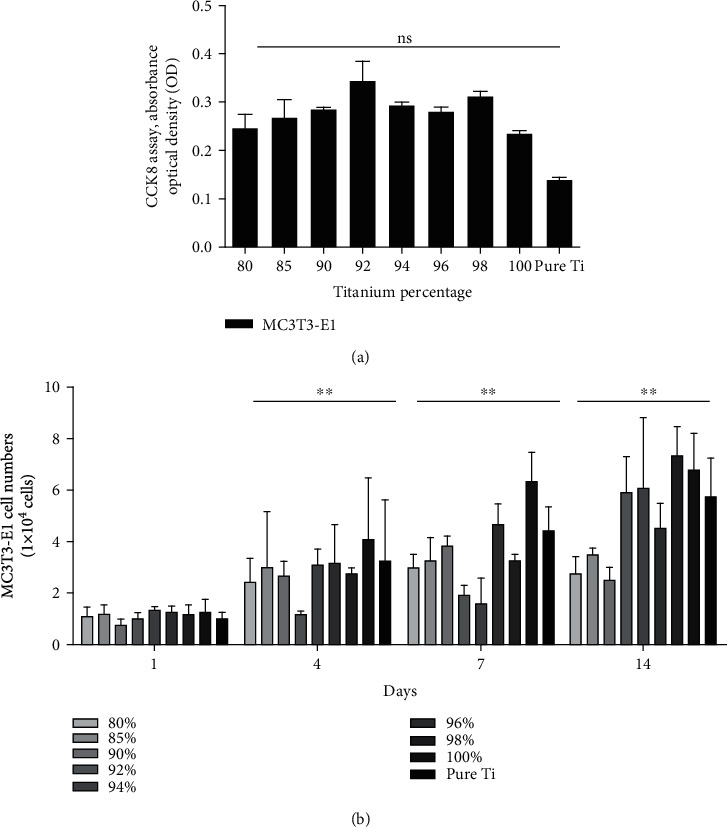
Cell viability in standard culture conditions. (a) CCK-8 assay revealed a stable profile in MC3T3-E1 cell cytotoxicity (*n* = 3, *P* = 0.11). (b) The 80% Ti VF group had the fewest number of cells, while the 100% Ti VF and pure Ti groups had the most number of cells (*n* = 3, ^∗∗^*P* < 0.0001).

The CCK-8 assay revealed that none of the Ti-HA groups exhibited cytotoxicity (Figure 4(a)). LDH assay was used to investigate whether the decreased metabolic activity of MC3T3-E1 cells after longer FGM exposure was related to the cytotoxic effects of the FGM (Table 2). After exposure of cells to composite for 48 h, the Ti-HA FGM showed no significant cytotoxicity towards MC3T3-E1 cells compared to the pure Ti control group (*P* = 0.812). After 120 h of exposure, the rate of cytotoxicity slightly increased (*P* = 0.920), but the difference was still not statistically different. The Ti-HA FGM with higher Ti VF exhibited more cell growth on the surfaces (Figure 4(b)).

### 3.3. Osteogenic Ability of the Ti-HA FGM

**Figure 5:**
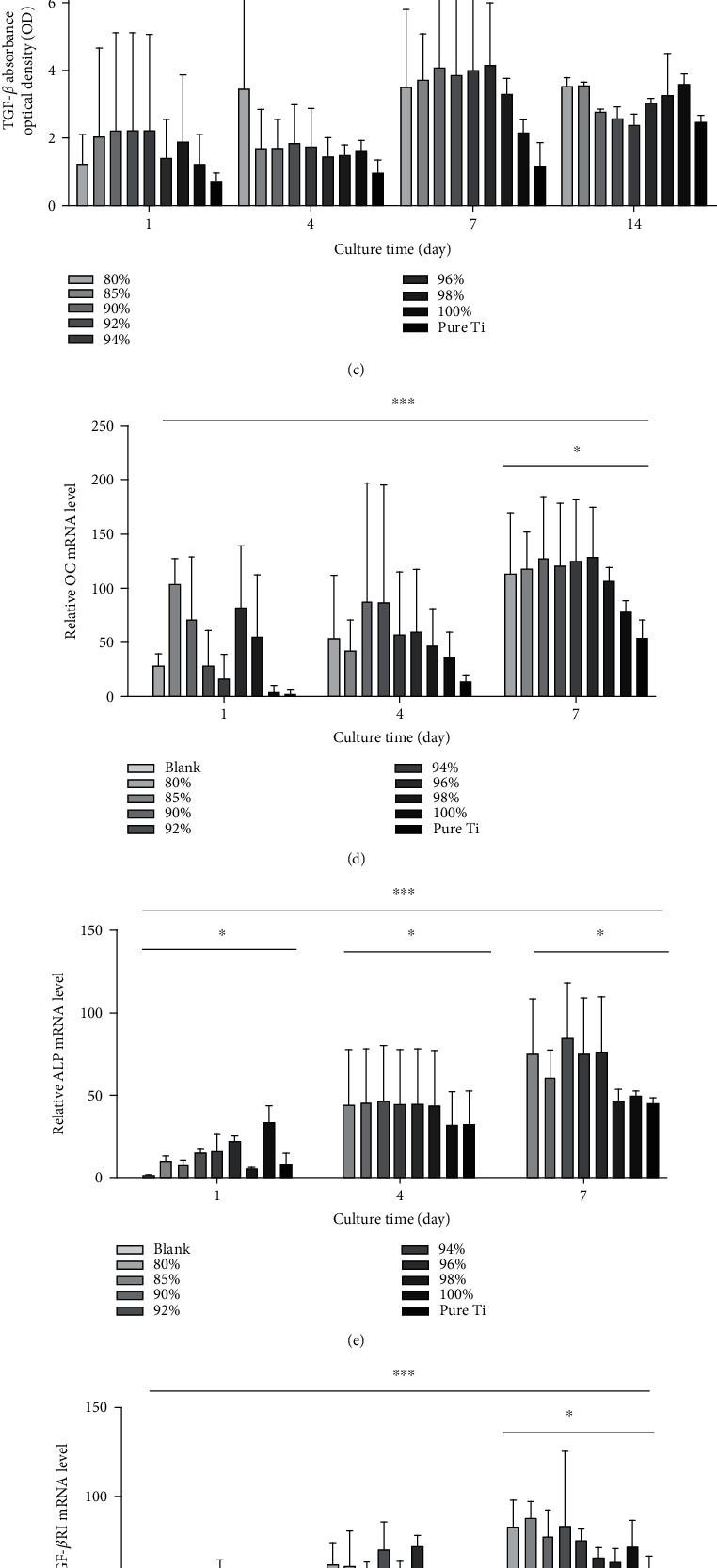
Cell osteogenic ability in standard culture conditions. (a) The OC levels over a 14-day culture period. (b) Over a 14-day period, the lowest ALP expression in the culture medium occurred in the alloys with the smallest Ti volume, but the expression levels in these groups were still higher than that in the Ti group (*n* = 3, *P* = 0.53). (c) TGF-*β*1 activity of the cells on Ti-HA increased steadily at 4 and 7 days. However, the difference between the groups at 1, 4, 7, and 14 days was not statistically significant (*n* = 3, *P* = 0.97, 0.89, 0.93, and 0.61, respectively). (d) OC transcript levels measured on days 1, 4, and 7 of culture showed a significant increase in time, with the highest activity on day 7. ^∗^*P* < 0.05, ^∗∗^*P* < 0.01, and ^∗∗∗^*P* < 0.001. The ALP transcript levels measured on days 1, 4, and 7 showed a significant increase with time, with the highest activity observed on day 7 (*n* = 3, *P* = 0.034). (e) The TGF-*β*RI mRNA level increased with the Ti VF (*n* = 3, *P* = 0.032). (f) The TGF-*β*RII mRNA level showed a slight increase (*n* = 3, *P* = 0.432).

As shown in Figure 5(a), osteocalcin (OC) expression was the highest at 14 days of culture, and the 94% composite showed the highest OC expression. The difference in the ALP expression between the three different groups did not significantly differ (Figure 5(b)). The Ti VF groups had similar TGF-*β*1 activity, which was comparable to that in the Ti group, for 14 days (Figure 5(c)). The relative *OC* mRNA transcript levels showed significant differences in expression at days 1, 4, and 7, with the highest expression occurring in the 80% Ti VF on day 7 (Figure 5(d)).

Thus, our findings revealed higher *ALP* (Figure 5(e)) and *TGF*-*βRI* (Figure 5(f)) transcript levels in the Ti-HA group than in the Ti group in 7 days, while there was no obvious change in the *TGF*-*βRII* level between the groups (Figure 5(g)). However, the differences in the relative mRNA expressions between the different materials were too small to be of statistical significance.

### 3.4. Biological Characterization of Ti-HA FGM Dental Implants

Figure 2(a) presents an illustration of the animal experiment design. Briefly, all mandibular premolars and first molars were extracted bilaterally during the first surgical session. Then, 12 weeks after the premolars were extracted, two implants were placed on each side of the mandibles of beagle dogs in the premolar region (Ti-HA FGM and Ti implants; length: 10 mm and diameter: 4.0 mm). After another 12 weeks of healing, the dogs were sacrificed. At 12 weeks after premolar extraction, two implants were placed on each side of the mandibles of beagle dogs in the premolar region as shown in Figure 2(b).

**Figure 6:**
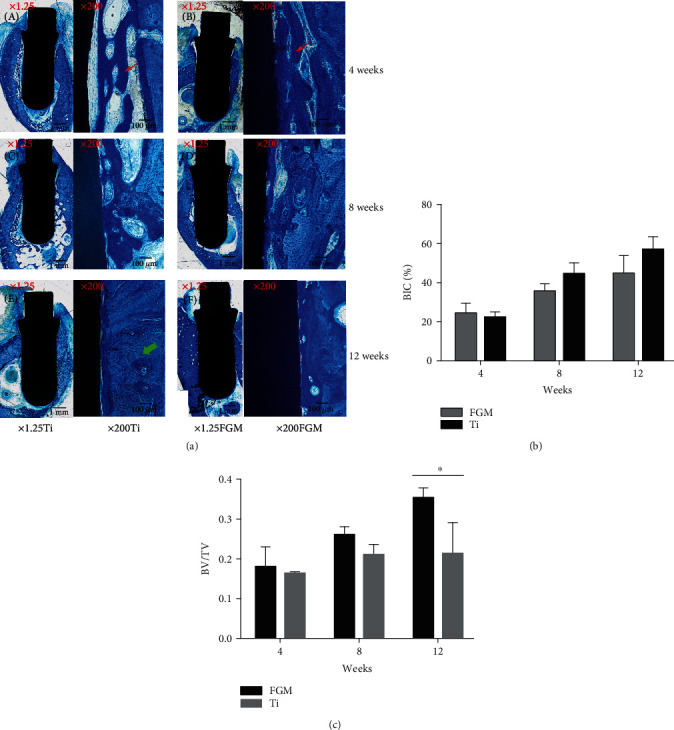
Histological analysis of Ti-HA FGM dental implants in vivo. (a) (A, B) Images illustrate that the calcified tissues around the implant were immature (white arrow) and surrounded by vascular spaces. (C, D) Images obtained at week 8 exhibit a higher level of contact between the implant and the denser osseous structure. (E, F) At week 12 in the Ti group and week 8 in the Ti-HA FGM group, mature osseous tissue can be seen gradually surrounding the implant surface (green arrow). (b) From weeks 4–12, bone-implant contact (BIC) (%) increased faster in the Ti-HA FGM group than in the Ti group (*n* = 3, *P* = 0.0504). (c) The Ti-HA FGM dental implant exhibited higher BV/TV values (*n* = 3, *P* < 0.05).

After the dogs received the Ti and Ti-HA FGM dental implants, the primary bone healing parameters were evaluated by micro-CT scan. After four weeks of healing, it can be seen by the CT scan results that the Ti and FGM implants were osseointegrated (Figure 2(c)). The newly formed bone volumes generated by the FGM implants were larger than those generated by the Ti implants. An X-ray test confirmed the results of the micro-CT scan. Successful osseointegration with no fibrous connective tissue between the implant and the bone was observed in both groups (Figure 6(a)). Histological analysis demonstrated that Ti-HA FGM had good osteogenic ability (Figure 6(a)). Bone formation at the periphery of the implant was clearly increased at 8 weeks compared with the image at 4 weeks. Haversian canals were observed in 12-week sections. No fibrous connective tissue was observed between the implant and the bone. The FGM dental implant had higher BV/TV values compared with Ti implants, and this difference was statistically significant (*n* = 3, *P* < 0.05), which showed the FGM group had more bone mineral proportion. However, the difference in BIC between the two groups was statistically nonsignificant, indicating that osseointegration between the two groups was comparable (Figures 6(b) and 6(c); *n* = 3, *P* = 0.0504). Unlike the cellular experiment, which was significant in the first week, the results of this experiment on dogs were inverse, indicating bone formation occurring over a longer period of time.

## 4. Discussion

Natural biomaterials are functionally graded. The Ti-HA FGM is regarded as the most promising replacement for lost dentin. Ti-HA FGM dental implants were fabricated, placed in canine jaws, and tested in our study. Ti-HA FGM contributes to the osteogenesis of the dental implant in vivo and in vitro. In our study, HA was incorporated into the implant body and not on the surface, effectively utilizing both the substances, and Ti-HA was fabricated as a compositional distribution profile.

The titanium alloys have been successfully used for dental implants [20, 21]. Watari et al. placed Ti-HA FGM dental implants in Wistar rats [22], and that research group subsequently improved the FGM [23]. Hedia used the finite element method to optimize the Ti-HA FGM dental implant [24]. In the present study, we modified the Ti-HA implant for achieving better osteogenesis. Bone formation at the implant-cell interface is a complex process. Higher protein adsorption ensures better cell attachment, which is the basis for osteogenesis. As observed on SEM, the surface morphology was rougher in FGM samples with higher HA content compared to pure Ti samples (Figure 3). Ti-HA FGM implants can be seen to have deep grooves extending into the alloy. The MC3T3-E1 cells migrated into the grooves, and numerous pseudopodia can be seen attached to the walls of the grooves. Using Ti implants with micrometer-range grooves might be one approach to optimize implant integration kinetics and stability in suboptimal clinical conditions. The present study showed that a rough and porous surface would enhance osteogenesis (Figure 3), which is consistent with the findings of a study reported previously [25].

The sandblasted and acid-etched surfaces are the most commonly used for the construction of dental implants [26]. However, commercial implants wherein the whole implants have the same hardness will result in bone resorption and atrophy around the implants, ultimately leading to implant mobility and failure [11, 27]. We effectively utilized HA and Ti to fabricate a FGM compositional distribution profile to avoid big elastic modulus differences. Ti-HA FGM exhibited better biocompatibility and bone formation than Ti (Figure 6).

Bone is essentially a vascularized matrix of organic proteins and inorganic calcium phosphate minerals [28]. The organic proteins primarily consist of fibrillar collagen type 1 fibers and mineralization-related proteins. Several extracellular matrix proteins are highly acidic, increasing speculation that they bind Ca^2+^ and initiate calcium phosphate synthesis to form an HA mineral phase with the Ca^2+^ from the surrounding medium [29, 30]. The data in this study demonstrate that the expression levels of OC, ALP, and TGF-*β*1 were lower in the specimens with lower HA and higher Ti. HA contained in the base might provide a good environment for calcification. OC, ALP, and TGF-*β*1 are proven biomarkers of osteogenic differentiation. ALP enzyme activity has been exhibited to increase in the early stages of osteogenic commitment, and *ALP* activity upregulation during osteogenic differentiation is related to the number of osteogenic committed progenitor cells [31]. Similarly, *TGF*-*β1* has been found to increase the presence of osteoblast differentiation markers (*Runx2*, *Opn*, and *Col1*) [32].

However, the differences in relative RNA expressions were too small to be significant in the present study. Surface topography exerts influence on the formed bone, and mineralized products can be guided by the metal surface topography. Our results indicated that the genes that encoded bone formation-related proteins were upregulated in the Ti-HA FGM.

Although BV/TV values are not related to osteointegration, they showed that the Ti-HA FGM had a higher bone mineral proportion. Micro-CT scan results and histomorphometric analysis results indicated that the Ti-HA FGM dental implants were superior to Ti implants in terms of biological characterization at the 12-week time point. The difference might have been greater if they had longer healing time and bite force loading for better stress distribution. The in vivo sample size is acceptable to draw a conclusion. We used six dogs and evaluated them at three time points, sacrificing two dogs at each time point, and this included the control. It was a trial model, and the *P* values were calculated to determine the required sample size. The number of animals was reduced to a minimum according to the “3Rs” [17]. Significant differences in the cellular experiment were revealed in the first week, while differences in the animal experiment became significant only in the third month; this might be because bone formation requires a longer duration.

We fabricated cylindrical implants using additive manufacturing technology. In the future, 3D printing technology may be used for FGM implant manufacturing. The present study may provide useful information in the improvement of present biomaterials and the discovery of future novel biomaterials.

## Figures and Tables

**Table 1:** Primers used in real-time PCR.

	Sense	Antisense
ALP	5′-CCCACGTCGATTGCATCTCT-3′	5′-AGTAAGGCAGGTGCCAATGG-3′
GAPDH	5′-GAGGACCAGGTTGTCTCCTG-3′	5′-GGATGGAATTGTGAGGGAGA-3′
TGF-*β*RI	5′-ATGGGCTTAGTATTCTGGGAA ATTGC-3′	5′-AGAATGACGAGAACATAACACTAG AGAC-3′
TGF-*β*RII	5′-TCCTGTTGACTGAGTTGCGATAAT G-3′	5′-GGTAGCAGTAGAAGATGATGATGA CAG-3′

**Table 2:** Cytotoxic effects of FGM on MC3T3-E1 cells evaluated by lactate dehydrogenase (LDH).

MC3T3-E1 cells	Incubation time	Volume fraction of titanium in FGM								
		80%	85%	90%	92%	94%	96%	98%	100%	Pure Ti
Cytotoxicity (%)	0 hours	0	0	0	0	0	0	0	0	0
	48 hours	0.43 ± 0.07	0.60 ± 0.10	0.67 ± 0.13	0.67 ± 0.23	0.77 ± 0.13	0.47 ± 0.13	0.77 ± 0.03	0.63 ± 0.17	0.60 ± 0.10
	120 hours	0.90 ± 0.20	1.50 ± 0.30	1.47 ± 0.23	1.67 ± 0.13	1.47 ± 0.13	1.80 ± 0.10	1.30 ± 0.20	1.17 ± 0.13	1.03 ± 0.17

LDH cytotoxicity assay was performed after 48 and 120 h of exposure. No cytotoxicity was observed with longer exposure to FGM.

## Data Availability

All data generated or analyzed during this study are included in this article. All data or models generated or used during the study are available from the corresponding author by request.

## References

[1] Mehta S. B., Banerji S. (2018). The restorative management of tooth wear involving the aesthetic zone. *British dental journal*.

[2] Jung W. M., Kim D. G., Yi Y. J., Park C. J., Cho L. R. (2007). Alveolar bone changes around the natural teeth opposing the posterior implants in mandible. *The Journal of Korean Academy of Prosthodontics*.

[3] Herrero-Climent M., López-Jarana P., Lemos B. F. (2020). Relevant design aspects to improve the stability of titanium dental implants. *Materials*.

[4] Chochlidakis K., Einarsdottir E., Tsigarida A. (2020). Survival rates and prosthetic complications of implant fixed complete dental prostheses: an up to 5-year retrospective study. *The Journal of prosthetic dentistry*.

[5] Demenko V., Linetskiy I., Nesvit K., Hubalkova H., Nesvit V., Shevchenko A. (2012). Importance of diameter-to-length ratio in selecting dental implants: a methodological finite element study. *Computer methods in biomechanics and biomedical engineering*.

[6] Merdji A., Aminallah L., Bachir Bouiadjra B. (2011). The mechanical behaviour of dental implant with stress barrier. *Zeszyty Naukowe Mechanika*.

[7] Achour T., Merdji A., Bouiadjra B. B., Serier B., Djebbar N. (2011). Stress distribution in dental implant with elastomeric stress barrier. *Materials & Design*.

[8] Robau-Porrua A., Pérez-Rodríguez Y., Soris-Rodríguez L. M., Pérez-Acosta O., González J. E. (2020). The effect of diameter, length and elastic modulus of a dental implant on stress and strain levels in peri-implant bone: a 3D finite element analysis. *Bio-medical materials and engineering*.

[9] Guillen-Romero L. D., Oropeza-Guzmán M. T., López-Maldonado E. A. (2019). Synthetic hydroxyapatite and its use in bioactive coatings. *Journal of applied biomaterials & functional materials*.

[10] Wang J., Zhang S., Sun Z., Wang H., Ren L., Yang K. (2019). Optimization of mechanical property, antibacterial property and corrosion resistance of Ti-Cu alloy for dental implant. *Journal of materials science & technology*.

[11] Lipphaus A., Witzel U. (2018). Finite-element syntheses of callus and bone remodeling: biomechanical study of fracture healing in long bones. *Anatomical record*.

[12] Asgharzadeh Shirazi H., Ayatollahi M. R., Asnafi A. (2017). To reduce the maximum stress and the stress shielding effect around a dental implant-bone interface using radial functionally graded biomaterials. *Computer methods in biomechanics and biomedical engineering*.

[13] Bordea I. R., Candrea S., Alexescu G. T. (2020). Nano-hydroxyapatite use in dentistry: a systematic review. *Drug metabolism reviews*.

[14] Tang X. J., Xing S. Z., Song X. L., Gui L., Chu C. L. (2005). Scanning electron microscopic study of titanium-hydroxyapatite, the functionally graded-material implanted in rabbits. *Chinese journal of plastic surgery*.

[15] Chenglin C., Jingchuan Z., Zhongda Y., Shidong W. (1999). Hydroxyapatite-Ti functionally graded biomaterial fabricated by powder metallurgy. *Materials Science and Engineering*.

[16] Livak K. J., Schmittgen T. D. (2001). Analysis of Relative Gene Expression Data Using Real-Time Quantitative PCR and the 2^−*ΔΔ* _C_^_T_ Method. *Methods*.

[17] Kilkenny C., Browne W. J., Cuthill I. C., Emerson M., Altman D. G. (2012). Improving bioscience research reporting: the ARRIVE guidelines for reporting animal research. *Osteoarthritis and cartilage*.

[18] Stokholm R., Isidor F., Nyengaard J. R. (2014). Histologic and histomorphometric evaluation of peri-implant bone of immediate or delayed occlusal-loaded non-splinted implants in the posterior mandible - an experimental study in monkeys. *Clinical Oral Implants Research*.

[19] Donath K., Breuner G. (1982). A method for the study of undecalcified bones and teeth with attached soft tissues. The Sage-Schliff (sawing and grinding) technique. *Journal of Oral Pathology*.

[20] Fiorillo L., D'Amico C., Campagna P., Terranova A., Militi A. (2020). Dental materials implant alloys: a X-ray fluorescence analysis on FDS76®. *Minerva Stomatologica*.

[21] Kim K. T., Eo M. Y., Nguyen T. T. H., Kim S. M. (2019). General review of titanium toxicity. *International Journal of Implant Dentistry*.

[22] Watari F., Yokoyama A., Saso F., Uo M., Kawasaki T. (1997). Fabrication and properties of functionally graded dental implant. *Composites Part B: Engineering*.

[23] Yokoyama A., Watari F., Miyao R. (2001). Mechanical properties and biocompatibility of titanium-hydroxyapatite implant material prepared by spark plasma sintering method.

[24] Hedia H. S. (2005). Design of functionally graded dental implant in the presence of cancellous bone. *Journal of biomedical materials research Part B, Applied biomaterials*.

[25] Yeo I. S. (2014). Reality of dental implant surface modification: a short literature review. *The open biomedical engineering journal*.

[26] Cervino G., Fiorillo L., Iannello G., Santonocito D., Risitano G., Cicciù M. (2019). Sandblasted and acid etched titanium dental implant surfaces systematic review and confocal microscopy evaluation. *Materials*.

[27] Bosshardt D. D., Chappuis V., Buser D. (2017). Osseointegration of titanium, titanium alloy and zirconia dental implants: current knowledge and open questions. *Periodontol 2000*.

[28] Kang Z., Zhang X., Chen Y., Akram M. Y., Nie J., Zhu X. (2017). Preparation of polymer/calcium phosphate porous composite as bone tissue scaffolds. *Materials Science & Engineering C*.

[29] Cui L., Houston D. A., Farquharson C., MacRae V. E. (2016). Characterisation of matrix vesicles in skeletal and soft tissue mineralisation. *Bone*.

[30] Di Filippo M. F., Dolci L. S., Albertini B. (2020). A radiopaque calcium phosphate bone cement with long-lasting antibacterial effect: from paste to injectable formulation. *Ceramics International*.

[31] Prins H.-J., Braat A. K., Gawlitta D. (2014). _In vitro_ induction of alkaline phosphatase levels predicts _in vivo_ bone forming capacity of human bone marrow stromal cells. *Stem cell research*.

[32] Zhao L., Jiang S., Hantash B. M. (2010). Transforming growth factor beta1 induces osteogenic differentiation of murine bone marrow stromal cells. *Tissue engineering Part A*.

